# Comparison of Glyphosate-Degradation Ability of Aldo-Keto Reductase (AKR4) Proteins in Maize, Soybean and Rice

**DOI:** 10.3390/ijms24043421

**Published:** 2023-02-08

**Authors:** Ronghua Chen, Siwei Wang, Yue Sun, Haiqing Li, Shuqing Wan, Fei Lin, Hanhong Xu

**Affiliations:** State Key Laboratory for Conservation and Utilization of Subtropical Agro-Bioresources/Key Laboratory of Natural Pesticide and Chemical Biology, Ministry of Education, South China Agricultural University, Guangzhou 510642, China

**Keywords:** glyphosate, aldo-keto reductase, glyphosate degradation, glyphosate-tolerant crops

## Abstract

Genes that participate in the degradation or isolation of glyphosate in plants are promising, for they endow crops with herbicide tolerance with a low glyphosate residue. Recently, the aldo-keto reductase (AKR4) gene in *Echinochloa colona* (*EcAKR4*) was identified as a naturally evolved glyphosate-metabolism enzyme. Here, we compared the glyphosate-degradation ability of theAKR4 proteins from maize, soybean and rice, which belong to a clade containing EcAKR4 in the phylogenetic tree, by incubation of glyphosate with AKR proteins both in vivo and in vitro. The results indicated that, except for OsALR1, the other proteins were characterized as glyphosate-metabolism enzymes, with ZmAKR4 ranked the highest activity, and OsAKR4-1 and OsAKR4-2 exhibiting the highest activity among the AKR4 family in rice. Moreover, OsAKR4-1 was confirmed to endow glyphosate-tolerance at the plant level. Our study provides information on the mechanism underlying the glyphosate-degradation ability of AKR proteins in crops, which enables the development of glyphosate-resistant crops with a low glyphosate residue, mediated by AKRs.

## 1. Introduction

Glyphosate is commonly used as a herbicide, owing to its advantages, including its high efficiency, low toxicity, and broad efficacy spectrum [1]. It mainly acts by competitively inhibiting 5-enolpyruvylshikimate-3-phosphate synthase (EPSPS) in the shikimate synthesis pathway, and sequentially disrupting the synthesis of plant-growth-regulating substances, such as aromatic amino acids, flavonoids and proteins, thereby leading to plant death [2,3]. Given its non-selective and deleterious effects, the use of glyphosate on many crops is limited. Thus, glyphosate-resistant crops are of great value as they would contribute the convenience of weeding and reduce the costs of rice production. Cope with glyphosate resistance has been generated via EPSPS-gene modification and applied in the field, leading to effective and safe weed management in modern agriculture [4,5].

Targeted and non-targeted resistance are two major ways to endow crops with resistance to a specific herbicide. EPSPS mutation or overexpression of variants can lead to a glyphosate-resistant phenotype in crops, and had been widely used in modern agriculture. Non-targeted resistance can be achieved by reducing the accumulated level of glyphosate in plants, such as by converting glyphosate to a low-toxicity substance or blocking its transport into its target [6,7,8,9]. Although EPSPS-based glyphosate resistance has been applied for many years, a concern regarding the residue of the herbicide after treatment remains [10]. Therefore, research interest is increasing in genes that participate in degradation or isolation of glyphosate in plants. Studies on glyphosate degradation at first mainly focus on microbes in the soil which have been contaminated by glyphosate. As early as 1992, it was identified that the glyphosate oxidoreductase (GOX) gene from a bacterium degrades glyphosate to aminomethyl phosphonic acid (AMPA) [11]. The *GOX* gene then transformed, along with *CP4* (a glyphosate-resistant *EPSPS* gene), into wheat and rape to obtained plants with high glyphosate resistance, as more glyphosate is metabolized to AMPA rapidly in transgenic lines [12,13]. Other glyphosate-metabolic genes were discovered that they encode glycine oxidase (GO) or D-amino acid oxidase (DAAO) participating in metabolizing glyphosate to yield AMPA and glyoxylate [14,15], thus endowing glyphosate resistance at the plant level [16,17]. However, to date, reports on native glyphosate-metabolizing enzymes in plants are still scarce. Several leguminous species may contain a GOX-like enzyme, but the mechanism of action is still unclear in plants [18,19,20]. Recently, plant endogenous enzymes that can detoxify glyphosate have emerged, all of which fall into the aldo-keto reductase (AKR) family [21,22].

The AKR superfamily is widespread in a variety of organisms, and encompasses 190 annotated proteins divided into 16 families by sequence identity [23,24]. Each AKR enzyme has three large loops on the surface of the α/β-barrel; moreover, owing to their high flexibility and low conservation, AKR enzymes have broad substrate specificity, ranging from the glucose, glucocorticoids and small carbonyl compounds to glutathione conjugates and phospholipid aldehydes, et al. [25,26]. For instance, AKR1D family members catalyze the Δ^4^-3-ketosteroids double-bond reduction to form 5β-dihydrosteroids [27,28,29]. AKR4C family members (AKR4C8, AKR4C9, AKR4C10 and AKR4C11) from *Arabidopsis thaliana* are involved in the detoxification of sugar-derived reactive carbonyls, and affect photosynthesis [30], as AKR4C7 from *Zea mays* induces the conversation of sorbitol to glucose [31]. The relationship between herbicide tolerance and AKRs is as follows: overexpression of *OsAKR4-1* in tobacco decreases the generation of toxic aldehydes generated by methyl viologen (paraquat) and recovers plant growth [32]. The transformation of butachlor to dicarboxylic acid and phenol was observed in *Escherichia coli* expressing AKR17A1 from *Anabaena* sp. [33,34]. In addition, the positive roles of PsAKR1, OsAKR4-1 and EcAKR4 in glyphosate degradation have been observed [21,22,35]. However, systematic studies in AKRs of plants that are regularly exposed to glyphosate are limited.

In this study, we performed bioinformatics analysis on genes with high homology with *EcAKR4*. The roles of these genes in glyphosate degradation were tested in *E. coli* cells, as well as in purified proteins. *OsAKR*-overexpressing rice lines were also constructed and subjected to glyphosate treatment to show their herbicide tolerance. The findings of our study pave the way to the AKR-based development of herbicide-resistant crops with low glyphosate residue.

## 2. Results and Discussion

### 2.1. Phylogenetic Analysis of AKR in Plants

**Figure 1:**
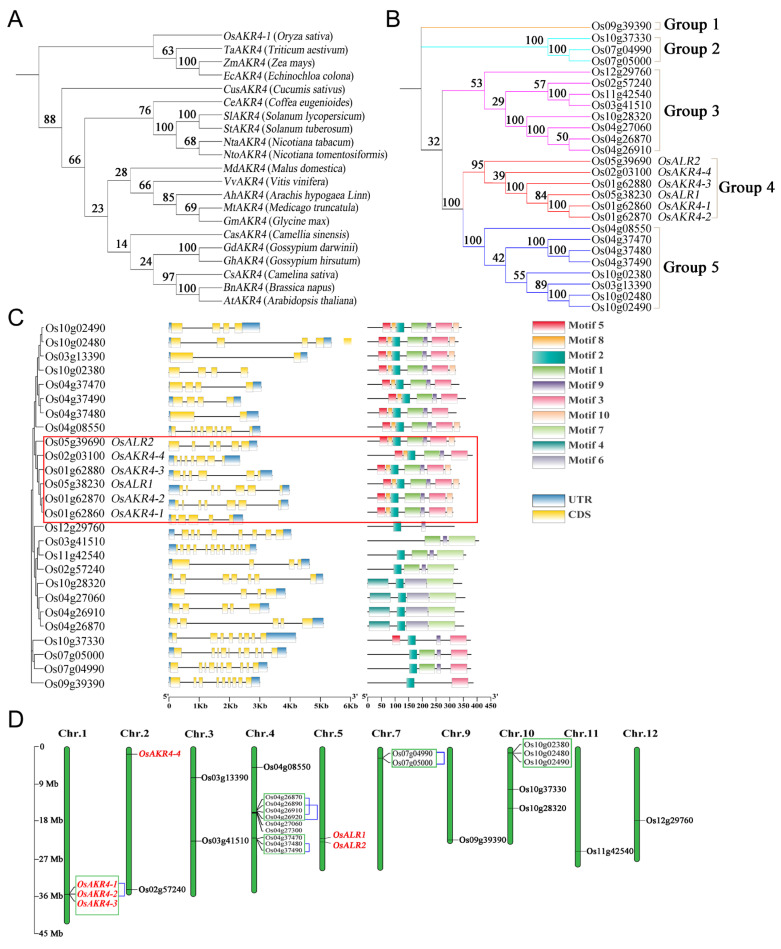
Bioinformatic analysis of AKR-family proteins. (**A**) Phylogenetic tree constructed from the amino acid sequences of the *AKR4* family in 21 plants. (**B**) Phylogenetic tree constructed from the amino acid sequences of OsAKRs. The sequences to be aligned were confirmed using SMART (http://smart.embl.de/smart, accessed on 5 March 2022) and NCBI CDD (www.ncbi.nlm.nih.gov/cdd/, accessed on 5 March 2022). (**C**) The gene structure and motif of OsAKRs. The scale at the bottom represents the number of nucleotides or amino acids. (**D**) Distribution of OsAKRs among the rice chromosomes. The genes in the green box represent tandem-duplicated genes. The large-fragment duplication event occurs in the genes connected with blue lines.

It has been demonstrated that the *EcAKR4* (*EcAKR4C10*) gene in *Echinochloa colona* confers glyphosate resistance to plants by encoding an enzyme that metabolizes glyphosate [22]. To investigate whether *AKR* genes in other plants also have similar functions, homologous genes in 21 crops were selected for phylogenetic-tree construction and evolutionary relationship analysis (Table S1). Nearest-neighbour analysis showed that the 21 *AKR* genes formed two distinct classes. Four genes from the Monocotyledoneae species, namely *OsAKR4-1*, *TaAKR4*, *ZmAKR4*, and *EcAKR4* belong to one clade, whereas the other 17 genes from the Dicotyledoneae species were grouped in one clade (Figure 1A). This result indicated that the *AKR4* genes underwent differentiation after the evolution of Monocotyledoneae and Dicotyledoneae [36].

The conserved domain of functional EcAKR4 with Pfam (PF00248) was applied to identify homologs of *AKRs* in rice by using SMART and NCBI CDD. A total of 29 *AKR* genes were identified in the rice whole genome. According to the results of phylogenetic-tree analysis, they were grouped into five subfamily clades, namely Groups 1 to 5. The *OsAKR4-1* genes were clustered in Group 4 with another five genes, and they were designated as *OsAKR4-2* (Os01g62870), *OsAKR4-3* (Os01g62880), *OsAKR4-4* (Os02g03100), *OsALR1* (Os05g38230), and *OsALR2* (Os05g39690), with 89%, 68%, 41%, 71%, and 46% amino-acid identity to *OsAKR4-1*, respectively (Figure 1B).

The structures of the *OsAKR4* genes were analyzed by comparing the genomic DNA sequence and the predicted cDNA sequence. The number of exons of the *OsAKR4* genes ranged from 2 to 12. Motif 2 was present in all OsAKR family members, except Os03g41510. The reported sites, namely Tyr-49, Lys-78, and Trp-112, which play critical roles in the interaction between glyphosate and EcAKR4 [22], were located at motifs 5, 8 and 2, respectively. All members in Group 4 contained the three conserved binding sites, implying that they may be involved in glyphosate metabolism, like the EcAKR4 functions (Figure 1C and Figure S1).

Gene-mapping analysis also showed that the *OsAKR4* genes were distributed unevenly on the rice genome. The largest gene cluster contained nine *OsAKRs* located on chromosomes 4 of rice with three duplication events, and the other two clusters were on chromosomes 1 and 10, which contained three genes for each. Three genes belonging to Group 4, that is, *OsAKR4-1*, *OsAKR4-2* and *OsAKR4-3*, were clustered on chromosome 1, with a large-fragment duplication event occurring between *OsAKR4-1* and *OsAKR4-2* (Figure 1D).

The six *OsAKRs* belonging to Group 4 were detected on chromosomes 1, 2, and 5. *OsAKR4-1*, *OsAKR4-2*, and *OsAKR4-3* were tandem-duplicated genes located on chromosome 1, whereas *OsALR1* and *OsALR2* were neighbours on chromosome 5, and *OsAKR4-4* was singly mapped to chromosome 2. These results implied that the OsAKR family arises from tandem duplication and gene duplications of a large chromosomal region. Most *OsAKRs* may originate from common ancestors, which accounts for the functional conservation.

### 2.2. AKR-Expressing E. coli Cells Acquire Tolerance to Glyphosate

**Figure 2:**
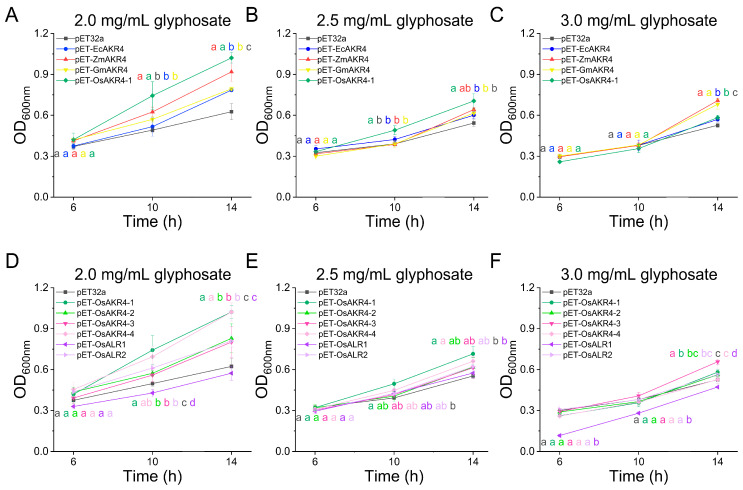
Growth of *Escherichia coli* BL21 expressing pET32a empty vector and pET32a-AKR in the presence of glyphosate. (**A**–**C**) *E. coli* growth curves plotted at OD_600nm_ influenced by *EcAKR4*, *ZmAKR4*, *GmAKR4* and *OsAKR4-1*. The cells were grown in a liquid medium containing 2.0 mg/mL (**A**), 2.5 mg/mL (**B**) or 3.0 mg/mL (**C**), of glyphosate. (**D**–**F**) *E. coli* growth curves plotted at OD_600nm_ influenced by *AKRs* from rice (*OsAKR4-1*, *OsAKR4-2*, *OsAKR4-3*, *OsAKR4-4*, *OsALR1* and *OsALR2*). The cells were grown in a liquid medium containing 2.0 mg/mL (**D**), 2.5 mg/mL (**E**), or 3.0 mg/mL (**F**), of glyphosate. Data are mean ± standard deviation. *n* = 3. (*p* < 0.05; Duncan’s multiple range tests).

To explore the *AKRs* that directly impart glyphosate tolerance in plants, we introduced *AKRs* into *E. coli* BL21 and examined its growth in the presence of glyphosate (Figure 2). The growth of *E. coli* harbouring the pET32a empty vector was impaired in media containing glyphosate, whereas the *E. coli* transfected with vectors harbouring *AKR4s* from *E. colona*, maize, soybean and rice exhibited stronger growth. Specifically, when cultured in 2.0 or 2.5 mg/mL glyphosate for 14 h, *E. coli* expressing *ZmAKR4* and *OsAKR4-1* showed the highest growth, while *ZmAKR4* or *GmAKR4*-expressing *E. coli* showed greater growth than the other strains when incubated with 3 mg/mL glyphosate (Figure 2A–C).

In addition, *E. coli* strains carrying rice *AKRs* (*OsAKR4-1*, *OsAKR4-2*, *OsAKR4-3*, *OsAKR4-4*, *OsALR1* and *OsALR2*) conferred varying degrees of sensitivity to glyphosate (Figure 2D–F). Moreover, pET32a-*OsAKR4-1* and *OsAKR4-4* carrying strains had remarkably higher growth than the pET32a empty vector under incubation with 2.0 or 2.5 mg/mL glyphosate. When the glyphosate concentration was increased to 3 mg/mL, *E. coli* strains expressing *OsAKR4-1* and *OsAKR4-3* exhibited high survival. In contrast, *E. coli* harbouring *OsALR1* exhibited an equivalent or even high-sensitivity phenotype to 3 mg/mL glyphosate, which was consistent with the finding that OsALR1 did not metabolize glyphosate [21]. Taken together, these findings indicated that *AKRs*, except *OsALR1*, endowed glyphosate tolerance in an *E. coli* system, with *ZmAKR4* leading to the highest tolerance among all *AKRs*, and *OsAKR4-1* exhibited the highest tolerance among the six *OsAKRs* in rice.

### 2.3. AKR Protein Expression and Substrate Activity

**Figure 3:**
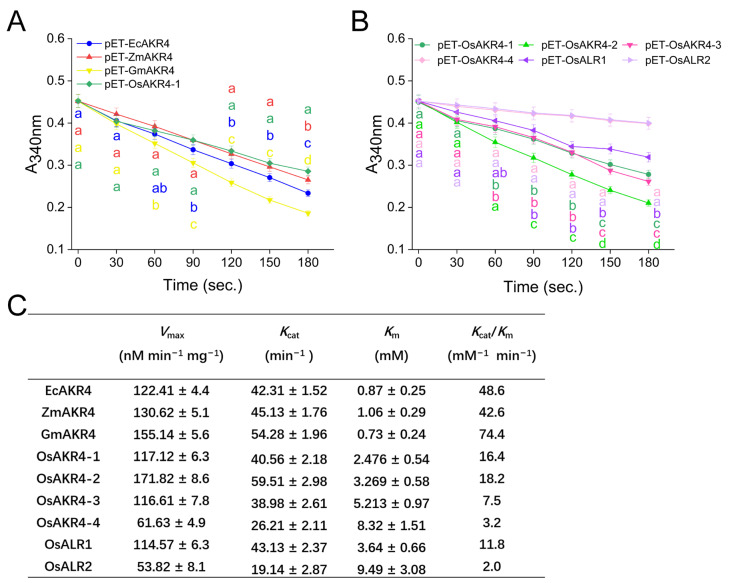
Activity of purified AKR proteins against benzaldehyde in vitro. (**A**,**B**) Enzyme activity of EcAKR4, ZmAKR4, GmAKR4, and OsAKR4-1 (**A**) along with OsAKRs (**B**) against 10 mM benzaldehyde. (**C**) Kinetic parameters of benzaldehyde metabolism by AKR enzymes. Data are mean ± standard deviation, *n* = 3.

AKR superfamilies typically use NADP^+^/NADPH as a co-factor to perform the catalytic reaction of aldehydes and ketones, sugar, and other specific substrates [26,37,38,39]. The changes in NADPH-absorbance level serve as an indicator for the metabolic activity of AKR proteins on their corresponding substrates. In this study, AKR proteins were purified using a Ni-NTA column from cell lysis of *E. coli* strains expressing AKRs at 16 °C, induced by 0.25 mM IPTG. The SDS-PAGE analysis revealed the successful expression of the proteins. The EcAKR4 protein showed a molecular mass of approximately 40 kD (kilo Dalton) [22], whereas the other AKRs were about 50 kD, consistent with the predicted molecular weight (Figure S2). The efficiency of AKR proteins in catalyzing the degradation of benzaldehyde and glyphosate was evaluated. With benzaldehyde as a substrate, GmAKR4 displayed the strongest degradation activity (*V*_max_ = 155.14 ± 5.6 nmol mg^−1^ min^−1^) among the AKR proteins tested (Figure 3A,C). Among the OsAKRs, OsAKR4-2 led to the largest reduction in A_340nm_ value, whereas OsAKR4-4 and OsALR2 did not cause any change (Figure 3B). Furthermore, enzyme kinetic curves were plotted for each AKR protein by conducting enzymatic reactions under a concentration gradient. GmAKR4 showed the lowest *K*_m_ value, suggesting that its benzaldehyde degradation ability was the highest among AKRs. Among OsAKRs, OsAKR4-1 and OsAKR4-2 exhibited high degradation activity on benzaldehyde. Moreover, the *K*_cat_/*K*_m_ ration was calculated for evaluating the catalytic efficiency and the result was similar to *K*_m_ result, in which GmAKR4 had the highest activity among all AKRs, and OsAKR4-1 and OsAKR4-2 in OsAKRs were the most efficient OsAKR for metabolizing benzaldehyde (Figure 3C and Figure S3).

**Figure 4:**
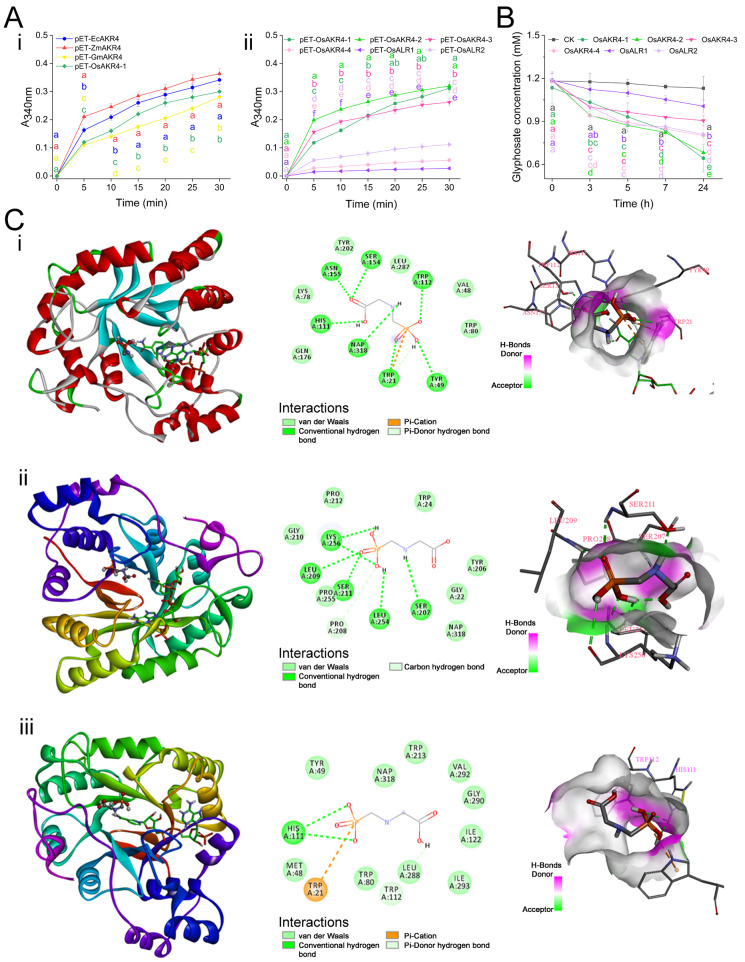
Comparison of the glyphosate-metabolism activities of AKR proteins. (**A**) Activity of purified EcAKR4, ZmAKR4, GmAKR4, OsAKR4-1 (**i**) and OsAKRs (**ii**) against glyphosate in vitro. Data are mean ± standard deviation, *n* = 3. (**B**) UPLC-MS/MS analysis of glyphosate metabolized by *E. coli*-expressed OsAKRs. (**C**) Molecular docking of NADP^+^ complexes with ZmAKR4 (**i**), GmAKR4 (**ii**), and OsAKR4-1 (**iii**) with glyphosate.

Unlike benzaldehyde to be hydrolyzed, glyphosate is typically oxidized by AKR proteins, with a similar degradation mechanism to that of 5α-dihydro-testosterone and xylitol [25,31,40]. This reaction induced the conversion of NADP^+^ to NADPH, leading to an increase in the A_340nm_ value. Evaluation of the enzyme reaction showed that with glyphosate as a substrate, the enzyme activity ranking was as follows: ZmAKR4 > EcAKR4 > OsAKR4-1 > GmAKR4 (Figure 4A(i)). ZmAKR4-expressing *E. coli* showed the highest glyphosate tolerance, as indicated by the detoxification of most of the glyphosate (Figure 2A–C). Previous studies revealed that ZmAKR4 preferred the conversion of sorbitol to glucose in the reaction (sorbitol + NADP^+^ ⇄ glucose + NADPH) [31,41]. Therefore, ZmAKR4 appeared to favour using NADP^+^ to oxidize glyphosate, rather than using NADPH to reduce benzaldehyde.

To further investigate the binding site between AKRs and glyphosate, molecular-docking analysis was performed. Glyphosate formed a conventional hydrogen bond with Trp-21, Try-49, His-111, Trp-112, Ser-154 and Asn-155 of ZmAKR4, as well as NADP^+^. Trp-21 also contacted with glyphosate by pi-cation and pi-donor hydrogen bond (Figure 4C(i)). GmAKR4 directly occupied the Ser-207, Pro-208, Leu-209, Ser-211, Leu-254, and Lys-256 amino acid sites (Figure 4C(ii)). For OsAKR4-1, its Trp-21, His-111, and Trp-112 residues directly contacted with glyphosate by pi-cation, the conventional hydrogen bond and the pi-donor hydrogen bond, respectively (Figure 4C(iii)). Moreover, Van Der Waals (VDW) interactions were also formed among glyphosate and the surrounding residues of ZmAKR4, GmAKR4 or OsAKR4-1. All of these above interactions contribute to the binding energy between glyphosate and these three AKR proteins.

### 2.4. OsAKR Proteins Are Involved in Glyphosate Metabolism

The glyphosate-degradation activities of OsAKRs were evaluated by comparing glyphosate-metabolism rates in AKR-expressing *E. coli*. The results showed that OsAKR4-1 and OsAKR4-2 had the highest reaction with glyphosate, whereas OsALR1 showed the lowest activity (Figure 4A(ii)).

The ability of OsAKRs to metabolize glyphosate was further evaluated by co-incubation analysis of glyphosate with the purified proteins in vitro. Glyphosate metabolism did not occur in the controls at all time points, while the glyphosate content decreased after incubation with OsAKR proteins. Compared with those in the control, glyphosate concentrations were 56.8%, 60.3%, 80.0%, 70.8%, 88.9%, and 71.6% after treatment for 24 h in *OsAKR4-1*, *OsAKR4-2*, *OsAKR4-3*, *OsAKR4-4*, *OsALR1* and *OsALR2*, respectively (Figure 4B). This result provided evidence that OsAKR4-1 and OsAKR4-2 had the highest glyphosate-metabolizing capacity among all OsAKRs. However, the degree of glyphosate degradation was low in the *OsALR1*-expressing *E. coli* system. A possible cause was that OsAKR4-1 could bind and catalyze glyphosate more efficiently than OsALR1, as demonstrated by molecular docking and dynamic analyses [21]. In addition, conserved Trp-21 was the binding site of glyphosate, but sequence alignment revealed that OsALR1 carried Ser in the Trp-21 site (Figure S4). Overall, ZmAKR4, OsAKR4-1 and OsAKR4-2 exhibited the highest glyphosate-degradation activity among the AKRs tested. They were also assembled in a subclade sharing, with *OsAKR4-2* sharing the most sequence identity (89%) with *OsAKR4-1* (Figure 1). This finding implies that the function of AKRs is conserved throughout its evolution.

### 2.5. The Response of OsAKRs in Rice Seedlings to Glyphosate Treatment

**Figure 5:**
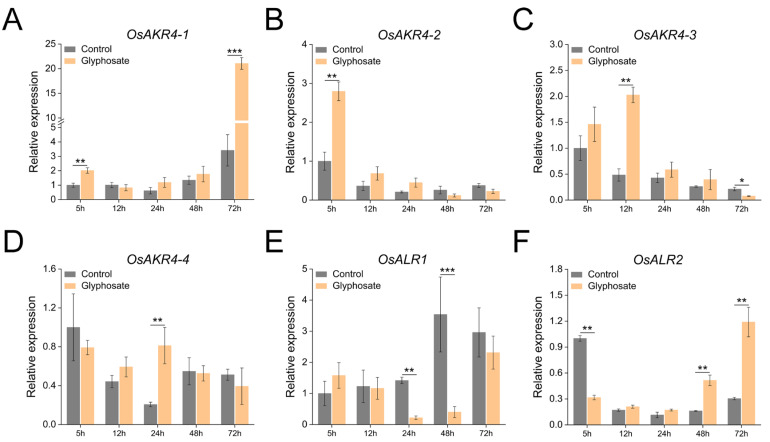
Transcription profiles of *OsAKRs* in response to glyphosate treatment. (**A**) for *OsAKR4-1*. (**B**) for *OsAKR4-2*. (**C**) for *OsAKR4-3*. (**D**) for *OsAKR4-4*. (**E**) for *OsALR1*. (**F**) for *OsALR2*. Two-week-old rice seedlings were sprayed with 13 mM glyphosate, and RNA was extracted from the shoots at 5, 12, 24, 48, and 72 h after treatment. Data are mean ± standard deviation, *n* = 3. (* *p* < 0.05, ** *p* < 0.01, *** *p* < 0.001; Duncan’s multiple range tests).

To investigate how the *OsAKRs* cooperate in response to glyphosate treatment, their expression patterns were monitored. The expression of most *OsAKRs* was induced by glyphosate treatment at a specific time point, except that of *OsALR1*, which showed no glyphosate-degradation function either in vivo or in vitro. The expression of both *OsAKR4-1* and *OsAKR4-2* in rice was induced by glyphosate treatment in a short time (5 h), with *OsAKR4-1* expression dramatically increasing at the 72 h time point, suggesting its critical role in the degradation of glyphosate. *OsAKR4-3* and *OsAKR4-4* expression was also upregulated to varying degrees at the mid-stage. Interestingly, a decrease in *OsALR1* expression was observed in the period of glyphosate spraying (Figure 5). These data indicate that OsAKR4-1 is a key enzyme for rice in glyphosate metabolism with working continuously.

**Figure 6:**
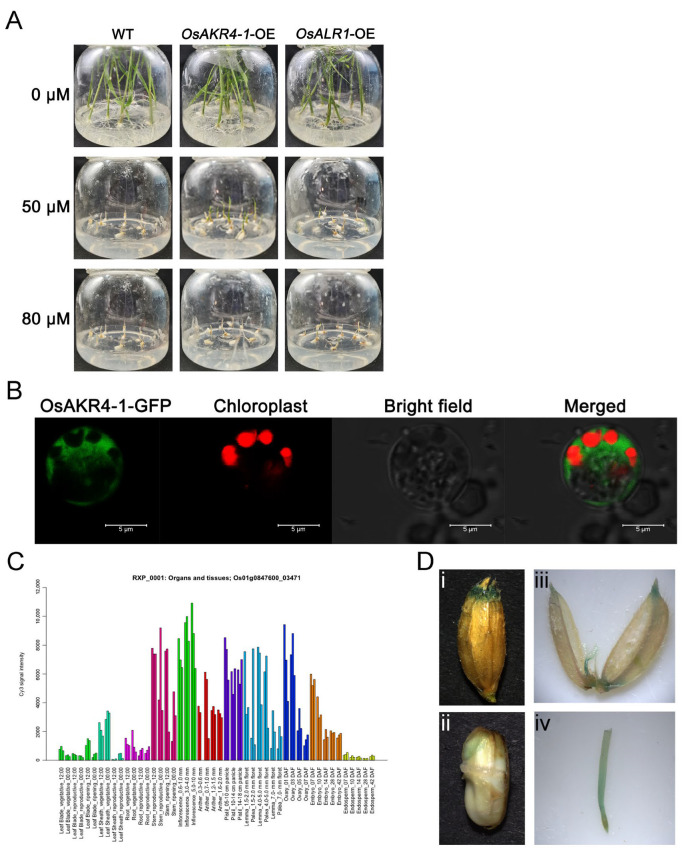
*OsAKR4-1* overexpressed in rice-seedling growth in glyphosate and its cellular and tissue localization. (**A**) Growth of germinated rice seedlings mediated by *OsAKR4-1* and *OsALR1* with 50 and 80 μM glyphosate. (**B**) Subcellular localization of OsAKR4-1 in rice protoplast; OsAKR4-1 fused with GFP was transiently co-expressed with chloroplast marker with mCherry in rice-sheath protoplasts. Scale bar = 5 µm. (**C**) Gene expression profiles of *OsAKR4-1* in various organs throughout the entire growth period, as quoted from RiceXPro (https://ricexpro.dna.affrc.go.jp, accessed on 18 July 2022). (**D**) Tissue-specific localization of *pOsAKR4-1*::GUS in transgenic rice, such as in the glume (**i**), seed (**ii**), lemma (**iii**), and root (**iv**).

To further address the role of OsAKR4-1 in response to glyphosate, we examined the phenotype of rice overexpressing *OsAKR4-1* and *OsALR1* under glyphosate stress (Figure S5). Glyphosate can lead to the inhibition of seedling growth and cause seedling wilting and browning. In our seed-germination experiment, the damage to the seedling was more pronounced in the wild type and *OsALR1*-OE than in the *OsAKR4-1*-OE under 50 μM glyphosate, whereas they showed similar inhibited growth in high-concentration glyphosate (80 μM) (Figure 6A). This result further validated the fact that OsAKR4-1 was a critical factor in metabolizing glyphosate but OsALR1 was not.

### 2.6. Localization of OsAKR4-1 in Rice

The signal of GFP-tagged OsAKR4-1 was localized in the cytoplasm, suggesting that the cytoplasm was the site of glyphosate metabolism by OsAKR4-1 (Figure 6B). Expression-pattern analysis showed that *OsAKR4-1* was mainly expressed in the stem, floral organs and seed ovary and embryo (Figure 6C); this finding resembles the result of the tissue-localization analysis. GUS-staining signals driven by *OsAKR4-1* promoter were detected at the glume, embryo, lemma and root (Figure 6D), illustrating that *OsAKR4-1* played an important role in seed germination and pollen development. Although OsAKR4-1 could degrade 43.9% of glyphosate, this ability was not as strong as that of EcAKR4 (92.5%) (Figure S6). It was observed that only OsAKR4-1 was not enough to endow excellent glyphosate resistance in rice (Figure 4B and Figure 6A). This observation was reasonable, as EcAKR4 conferred glyphosate resistance only in plants harboring the EPSPS target mutation [42]. Therefore, it is reasonable to infer that *OsAKR4-1* can be coupled with the target gene to provide herbicide resistance in rice for commercial production. The advantages of such a strategy lie in improving glyphosate resistance while reducing glyphosate residue in crops [5,43,44].

## 3. Materials and Methods

### 3.1. Sequence Homology among Different Plants

To study the relationship of the AKR family among plants that are often exposed to glyphosate, the amino acid sequences of 21 plants were retrieved from NCBI (https://www.ncbi.nlm.nih.gov/, accessed on 5 March 2022) for phylogenetic analysis. The plants included *Arabidopsis thaliana* (AT2G37790), *Arachis hypogaea* Linn (LOC112727795), *Brassica napus* (LOC106451053), *Camelina sativa* (LOC104785711), *Camellia sinensis* (LOC114297399), *Coffea eugenioides* (LOC113772474), *Cucumis sativus* (LOC101209936), *Echinochloa colona* (MK592097), *Glycine max* (LOC100802323), *Gossypium darwinii* (TYH22311), *Gossypium hirsutum* (LOC107888724), *Malus domestica* (LOC103450273), *Medicago truncatula* (LOC25501528), *Nicotiana tabacum* (LOC107767146), *Nicotiana tomentosiformis* (LOC104097840), *Oryza sativa* (LOC4327475), *Solanum lycopersicum* (LOC101256514), *Solanum tuberosum* (LOC102593301), *Triticum aestivum* (KAF7024607.1), *Vitis vinifera* (LOC100254427) and *Zea mays* (LOC100037812). A total of 21 sequences were aligned, using ClustalW version 2.1. Next, the sequences were grouped through neighbour-joining analysis with 1000 bootstrap replicates using MEGA version 7.0. Phylogenetic trees were drawn using iTOL (https://itol.embl.de/, accessed on 5 March 2022).

The OsAKR4-1 (LOC4327475) protein sequence from rice was submitted to the Pfam (http://pfam.xfam.org, accessed on 5 March 2022) to construct a hidden Markov model (HMM), and matching sequences were obtained using the hmmsearch program. The representative sequences were confirmed using SMART (http://smart.embl.de/smart, accessed on 5 March 2022) and NCBI CDD (www.ncbi.nlm.nih.gov/cdd/, accessed on 5 March 2022) and then used for phylogenetic analysis, as mentioned before. Moreover, the protein sequences of OsAKRs were submitted to the MEME database (http://meme.nbcr.net/meme/intro.html, accessed on 5 March 2022) to find conserved motifs, searching for up to 10 motifs. Comparative analysis of exon–intron gene structures for OsAKRs was conducted by GSDS (http://gsds.cbi.pku.edu, accessed on 5 March 2022). Finally, the physical map of the chromosome was visualized using the MapChart software (ADInstruments, Newcastle, Australia).

### 3.2. Cloning Procedure and Plasmid Construction

*EcAKR4*, *ZmAKR4*, *GmAKR4* and *OsAKRs* (*OsAKR4-1*, *OsAKR4-2*, *OsAKR4-3*, *OsAKR4-4*, *OsALR1* and *OsALR2*) were selected for further analysis. The coding sequence (CDS) of these candidate AKR genes was amplified (Table S2) using the cDNA from plant leaves as a template and then cloned into the *pEASY*-Blunt cloning vector (Transgen, Beijing, China) following the manufacturer’s instruction. However, the CDS of *GmAKR4* and *OsALR1* could not be amplified, and thus were chemically synthesized by BGI (BGI, Beijing, China). Subsequently, the target genes were sub-cloned into the expression vector pET32a, using the in-fusion method (Takara, Dalian, China). All recombinant vectors were sequenced, to confirm that the inserts were correct.

### 3.3. Expression and Purification of AKRs in E. coli

The resulting pET32a-*EcAKR4*, *ZmAKR4*, *GmAKR4*, and *OsAKRs* constructs were transferred into the *E. coli* BL21 as the expression host. The cells were grown on an LB solid medium containing ampicillin (Amp) for 10 h, at 37 °C. The colonies were then cultured in an LB liquid medium (Amp^+^), with shaking at 200 rpm. When the OD_600_ reached 0.6–0.8, IPTG was added at a final concentration of 0.25 mM. Cells were grown at 16 °C for 16 h to induce the expression of target proteins. Next, the cells were harvested by centrifugation at 6000× *g* at 4 °C for 10 min, resuspended in a lysis buffer (25 mM Tris-HCl pH 7.5, 300 mM NaCl, and 20 mM imidazole), and then disrupted using a high-pressure cell cracker at 1500 bar. Cell debris was discarded by centrifugation at 11,000× *g* at 4 °C for 30 min. The supernatant was applied to an Ni-NTA column that had been equilibrated with five column volumes of lysis buffer. Other proteins were removed using wash buffer (25 mM Tris-HCl pH 7.5, 300 mM NaCl, and 70 mM imidazole), and bound proteins were eluted using an elution buffer (20 mM Tris-HCl pH 8.0, 300 mM NaCl, and 300 mM imidazole). The purity of the protein was determined by 12% SDS-PAGE analysis and then stored at −80 °C, with glycerin added.

### 3.4. Comparative-Growth Test of Glyphosate in E. coli

Briefly, *E. coli* containing the pET32a gene were shake-cultured in an LB medium (Amp^+^) for 4 h, and IPTG was added at a final concentration of 1 μM, followed by the addition of glyphosate at various concentrations (2, 2.5 and 3 mg/mL). The cultures were then incubated with shaking at 180 rpm and 37 °C. The OD_600_ of each culture was monitored at 6, 10 and 14 h for plotting the OD_600_-growth-time function.

### 3.5. Measurement of Enzyme Activity in E. coli

AKR activities in *E. coli* were measured as described previously, with modification [23]. For experiments using benzaldehyde as a substrate, the reaction mixture (1 mL) contained 50 mM phosphate buffer (pH 7.4), 0.1 mM NADPH, 10 mM β-mercaptoethanol, 100 μg purified protein and 10 mM benzaldehyde. In the enzyme kinetic analysis, the concentration of benzaldehyde in the reaction mixture was set to 2.5, 5.0, 7.5, 10, 15 and 20 mM. For experiments using glyphosate as a substrate, the reaction mixture (1 mL) contained 75 mM Tris-HCl buffer (pH 8.6), 0.1 mM NADP^+^, 10 mM β-mercaptoethanol, 20 μg purified protein, and 0.5 mM glyphosate. All reactions proceeded at 25 °C for different durations (up to 3 min for benzaldehyde and up to 30 min for glyphosate). The enzyme activity was confirmed according to the absorbance of NADPH at 340 nm, as measured using a biophotometer (Eppendorf, Germany).

### 3.6. Degradation of Glyphosate by E. coli

The purified OsAKR proteins were used for catalyzing the glyphosate degradation. In the reaction mixture, 20 μg purified protein was cultured with excessive 0.1 mM NADP^+^, 0.5‰ Tween 80 and 1.48 mM glyphosate in the 75 mM Tris-HCl buffer at pH 6.8 and 35 °C for different durations (0, 3, 5, 7, and 24 h). Mixtures without AKR protein were treated as controls. The UPLC-MS/MS analysis was performed to determine the content of derivatized glyphosate in the mixture.

### 3.7. Molecular Docking of AKRs with Glyphosate

The spatial structures of ZmAKR4, GmAKR4, and OsAKR4-1 were downloaded from the AlphaFold Protein Structure Database (https://alphafold.com/, accessed on 3 July 2022) [45,46]. The NADP^+^-bound AKRs were used for molecular docking with glyphosate. The binding pocket of ZmAKR4, GmAKR4, and OsAKR4-1 were set to be center x/y/z = 8.487/−0.624/−0.572, 5.308/6.165/5.503 and 5.176/9.278/−2.137, respectively. Docking was performed in the AutoDock Vina, followed by preparation of the three-dimensional graphics using Discovery Studio.

### 3.8. Measurement of Glyphosate Using UPLC-MS/MS

The samples were reacted with 9-fluorenylmethoxycarbonyl chloride (Fmoc-Cl) (1.0 g/L in acetone) in a sodium borate buffer (50.0 g/L, pH 9) at 37 °C for 10 h, to generate Fmoc-derivative products. UPLC-MS/MS analysis was performed using a Xevo TQD mass spectrometer (Waters, Milford, MA, USA) interfaced with ACQUITY UPLC^®^ H-Class system. The samples were separated by an ACQUITY UPLC BEH C18 (2.1 × 100 mm, 1.7 μm, Waters), with acetonitrile and 2 mM ammonium acetate water with 0.1% formic acid as the mobile phase. The following instrument parameters were used: desolvation temperature, 550 °C; cone voltage, 25 V; capillary voltage, 500 V. The MS/MS ion transitions and collision energy for compounds were as follows: glyphosate, ESI^+^, *m*/*z* 392.1-88 (25 eV), 170 (12 eV), and 214 (12 eV).

### 3.9. Gene-Expression Analysis Using qRT-PCR

Rice seedlings were grown in modified Hoagland nutrient solutions for 2 weeks, and later sprayed with 13 mM glyphosate. RNA was isolated from the shoots at 5, 12, 24, 48 and 72 h after treatment, and subjected to qRT-PCR analysis for monitoring the expression of *OsAKRs*.

### 3.10. Glyphosate-Tolerance Seed-Germination Assay

The promoter sequences of *OsAKR4-1* and *OsALR1* were amplified by PCR and cloned into the pCAMBIA1300-35S vector with 35S promoter. Then they were transformed into Zhonghua11 by *Agrobacterium tumefaciens*-edited transformation to generate *OsAKR4-1*-OE and *OsALR1*-OE lines, which were confirmed by gene-expression-level analysis, using qRT-PCR. For observing the resistance to glyphosate, the seeds of *OsAKR4-1*-OE and *OsALR1*-OE lines were germinated on MS medium supplemented with 50 or 80 μM glyphosate for 2 weeks, and photographed.

### 3.11. Subcellular Localization of OsAKR4-1

To detect the localization of OsAKR4-1, the OsAKR4-1-GFP plasmid was transfected into the rice protoplast for screening the fluorescence signal, using a Leica SP8 confocal laser scanning microscope as the parameter mentioned previously [47].

### 3.12. Histochemical Determination of GUS Activity

Germinating rice seeds, the root, stem and leaf of 2-week-old seedlings, and rice florets at the flowering stage were stained using a GUS solution kit (Coolaber, Beijing, China). In brief, all tissues were incubated overnight at 37 °C and then cleared in the 70% ethanol. The GUS-staining results were observed using a stereoscope, as described previously [47].

## 4. Conclusions

Glyphosate is an efficient broad-spectrum herbicide widely used in agricultural production. The cultivation of transgenic glyphosate-resistant crops has significantly increased the easy use of glyphosate, in which the crops endowed metabolic resistance, is being considered. Previous studies have shown that AKR in *E. colona* has the function of degrading glyphosate. In this study, we compared the effectiveness of AKRs from maize, soybean, and rice with that of EcAKR4 in metabolizing glyphosate. All these proteins have emerged as novel enzymes for glyphosate degradation, with ZmAKR4 showing the highest activity. Moreover, OsAKR4-1 and OsAKR4-2 exhibited excellent glyphosate-metabolizing capacity (among six OsAKRs), and *OsAKR4-1* provided continuous response to glyphosate in plants so, that the rice with overexpressed *OsAKR4-1* had a degree of resistance to glyphosate. Thus, we demonstrate these genes as potential candidates for cultivating glyphosate-resistant crops with low glyphosate residue.

## Data Availability

Not applicable.

## Supplementary Materials

The following supporting information can be downloaded at https://www.mdpi.com/article/10.3390/ijms24043421/s1.
