# Supplementary material for: Comparison of Glyphosate-Degradation Ability of Aldo-Keto Reductase (AKR4) Proteins in Maize, Soybean and Rice
Source: Int J Mol Sci. 2023 Feb 8;24(4):3421. doi: 10.3390/ijms24043421 (PMC9966811; doi:10.3390/ijms24043421)
Supplement: Supplementary file 1 [file ijms-24-03421-s001.zip › Table S1.pdf]

Table S1. Information of *OsAKRs* genes in rice genome.

RGAP-ID [a]	Chromosome Location [a]	gDNA Length [b]	Protein Length [b]	MW (Da) [c]	pI [c]
Os12g29760	12	2439	317	34229.9	5.22
Os11g42540	11	2878	359	39976.2	6.74
Os10g37330	10	4188	376	41141.4	9.76
Os10g28320	10	5083	344	37642.9	6.01
Os10g02490	10	2997	343	38024.2	5.71
Os10g02480	10	5359	331	37016.3	6.71
Os10g02380	10	4565	321	35577.6	5.11
Os09g39390	9	2998	386	42216.9	7.89
Os07g05000	7	3873	378	40600.9	8.54
Os07g04990	7	3245	377	40552.8	7.1
Os05g39690	5	3015	319	35589.5	6.79
Os05g38230	5	3405	335	37674.2	8.22
Os04g37490	4	3047	358	39541.2	8.61
Os04g37480	4	2370	324	36123.1	6.00
Os04g37470	4	2601	334	36501.9	7.81
Os04g27300	4	548	120	12391.9	11.04
Os04g27060	4	3841	356	38273.2	5.85
Os04g26920	4	3193	159	17011.4	8.09
Os04g26910	4	3301	352	38233.2	6.43
Os04g26890	4	767	179	18557.4	9.50
Os04g26870	4	5098	351	38118.5	6.98
Os04g08550	4	2951	338	35765.5	6.57
Os03g41510	3	4031	406	45663.5	8.18
Os03g13390	3	2785	319	35421.3	7.44
Os02g57240	2	4637	329	36449.2	7.49
Os02g03100	2	2909	383	42559.3	8.16
Os01g62880	1	2341	305	33435.1	6.17
Os01g62870	1	3972	312	34634.4	7.06

[a] The information of RGAP-ID and chromosome location were from RGAP database (http://rice.uga.edu/index.shtml), [b] the information of gDNA length and protein length were from NCBI database (https://www.ncbi.nlm.nih.gov), [c] the information of protein molecular weight and isoelectric point were from Expasy (https://www.expasy.org/).
