# Supplementary material for: Comparison of Glyphosate-Degradation Ability of Aldo-Keto Reductase (AKR4) Proteins in Maize, Soybean and Rice
Source: Int J Mol Sci. 2023 Feb 8;24(4):3421. doi: 10.3390/ijms24043421 (PMC9966811; doi:10.3390/ijms24043421)
Supplement: Supplementary file 1 [file ijms-24-03421-s001.zip › Table S2.pdf]

Table S2. Primers sequences used in this study.

Name	Primer Sequence
TA-ZmAKR4-F	ATGGCGCGGCACTTCGTGCTCA
TA-ZmAKR4-F	TTAAAGTTCACCATCCCAGAGC
TA-EcAKR4-F	ATGGCGAGGCACTTCGTGCT
TA-EcAKR4-R	CTAAAGTTCACCGTCCCAGAGC
TA-OsAKR4-1-F	ATGGCGAAGCATTTCGTGCTCAACA
TA-OsAKR4-1-R	AGCTCTGGGACGGCGAAATTTAG
TA-OsAKR4-2-F	ACCACCGCAACGGAAACAC
TA-OsAKR4-2-R	GGCACTGACGGAACTCCTAAAT
TA-OsAKR4-3-F	ATGGCCACCCACTTCACGCT
TA-OsAKR4-3-R	TTAGATTTCTCCATCAAAAAGCTCC
TA-OsAKR4-4-F	ATGGCCATCACAGTGTCTATCT
TA-OsAKR4-4-R	TCAAGCAAACAGGTCGATTCCC
TA-OsALR2-F	ATGGCGAGTGCCAAGGCGAT
TA-OsALR2-R	TTAGACCTCGTTATCCCAGACC
pET32a-Gm242-F	CCATGGCTGATATCGGATCCATGTCGAACGATATTGGATTCTTCG
pET32a-Gm242-F	GTGGTGCTCGAGTGCGGCCGCTTAGATTTCACCATCCCATAATTCC
pET32a-ZmAKR4-F	CCATGGCTGATATCGGATCCATGGCGCGGCACTTCGTGCTCA
pET32a-ZmAKR4-F	GTGGTGCTCGAGTGCGGCCGCTTAAAGTTCACCATCCCAGAGC
pET32a-EcAKR4-F	CCATGGCTGATATCGGATCCATGGCGAGGCACTTCGTGCT
pET32a-EcAKR4-R	GTGGTGCTCGAGTGCGGCCGCCTAAAGTTCACCGTCCCAGAGC
pET32a-OsAKR4-1-F	CCATGGCTGATATCGGATCCATGGCGAAGCATTTCGTGCTCAACA
pET32a-OsAKR4-1-R	GTGGTGCTCGAGTGCGGCCGCAGCTCTGGGACGGCGAAATTTAG
pET32a-OsAKR4-2-F	CCATGGCTGATATCGGATCCACCACCGCAACGGAAACAC
pET32a-OsAKR4-2-R	GTGGTGCTCGAGTGCGGCCGCGGCACTGACGGAACTCCTAAAT
pET32a-OsAKR4-3-F	CCATGGCTGATATCGGATCCATGGCCACCCACTTCACGCT
pET32a-OsAKR4-3-R	GTGGTGCTCGAGTGCGGCCGCTTAGATTTCTCCATCAAAAAGCTCC
pET32a-OsAKR4-4-F	CCATGGCTGATATCGGATCCATGGCCATCACAGTGTCTATCT
pET32a-OsAKR4-4-R	GTGGTGCTCGAGTGCGGCCGCTCAAGCAAACAGGTCGATTCCC

Name	Primer Sequence
pET32a-OsALR1-F	CCATGGCTGATATCGGATCCATGGCAGGCCGAGCCGGGCGTC
pET32a-OsALR1-R	GTGGTGCTCGAGTGCGGCCGCTTAGATTTCGCCATCCCAAAGC
pET32a-OsALR2-F	CCATGGCTGATATCGGATCCATGGCGAGTGCCAAGGCGAT
pET32a-OsALR2-R	GTGGTGCTCGAGTGCGGCCGCTTAGACCTCGTTATCCCAGACC
ACTIN-F	TGCTATGTACGTCGCCATCCAG
ACTIN-R	AATGAGTAACCACGCTCCGTCA
OsAKR4-1-qRTPCR-F	AACACTGAAGACTACATACCACCT
OsAKR4-1-qRTPCR-R	ACTTACACCAATGGCACGAGA
OsAKR4-2-qRTPCR-F	CAGCTGTTGACCAGGTGGAG
OsAKR4-2-qRTPCR-R	CTAGCGGCGAGTATGCAGAAA
OsAKR4-3-qRTPCR-F	AGGGTACCGGCACATAGACT
OsAKR4-3-qRTPCR-R	GAGGTCGCTGCACCATATCT
OsAKR4-4-qRTPCR-F	ACCCGTCATACTGGAGTTGG
OsAKR4-4-qRTPCR-R	TGCTAATCCCAATGCTGCGA
OsALR1-qRTPCR-F	AGGAATCCACCTCTCAGCGT
OsALR1-qRTPCR-R	TCTGAATCCCCCACCGTAGA
OsALR2-qRTPCR-F	GTGCTCATCAAGTGGGCTCT
OsALR2-qRTPCR-R	CTGTCAGGACTCGCTTCTCA
