# Supplementary figures and images for: Comparison of Glyphosate-Degradation Ability of Aldo-Keto Reductase (AKR4) Proteins in Maize, Soybean and Rice
Source: Int J Mol Sci. 2023 Feb 8;24(4):3421. doi: 10.3390/ijms24043421 (PMC9966811; doi:10.3390/ijms24043421)

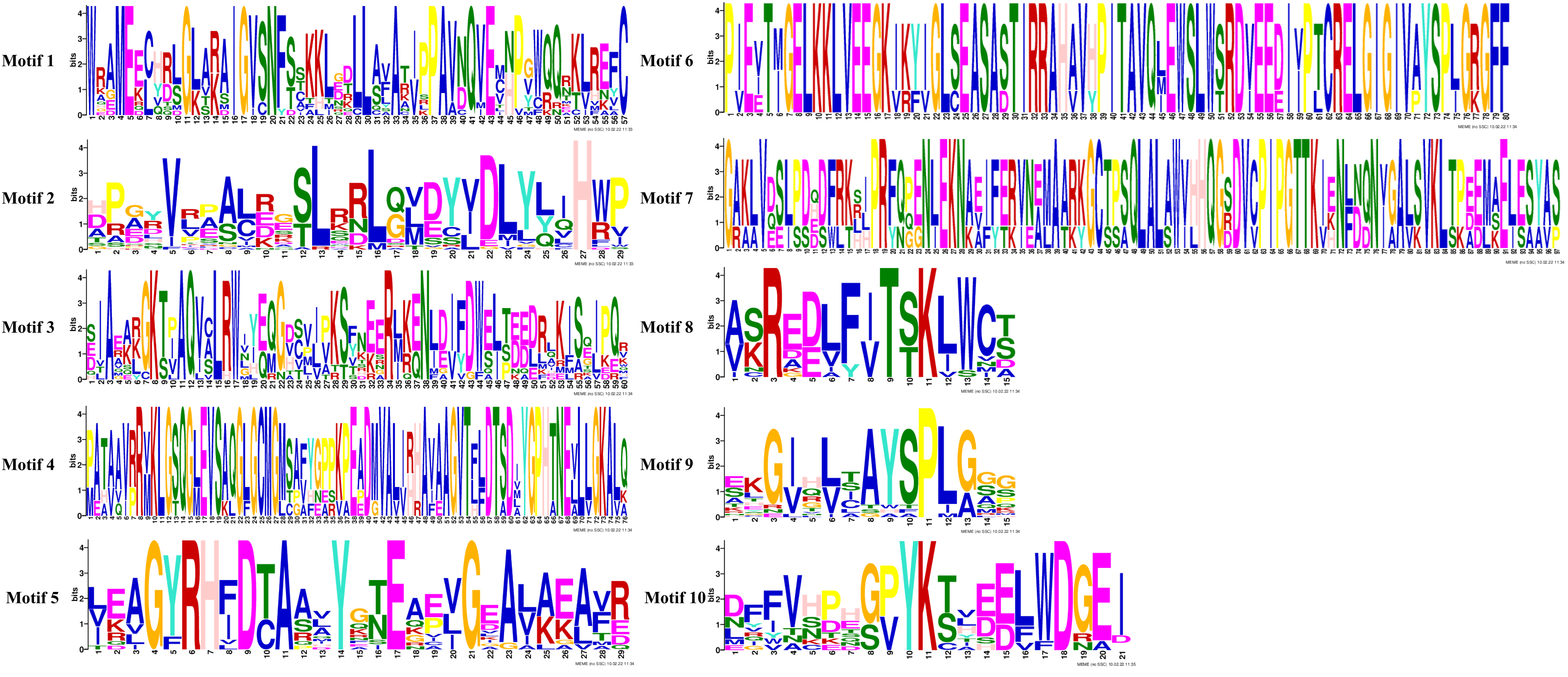

Supplement: Supplementary file 1 [file ijms-24-03421-s001.zip › Figure S1.tif]

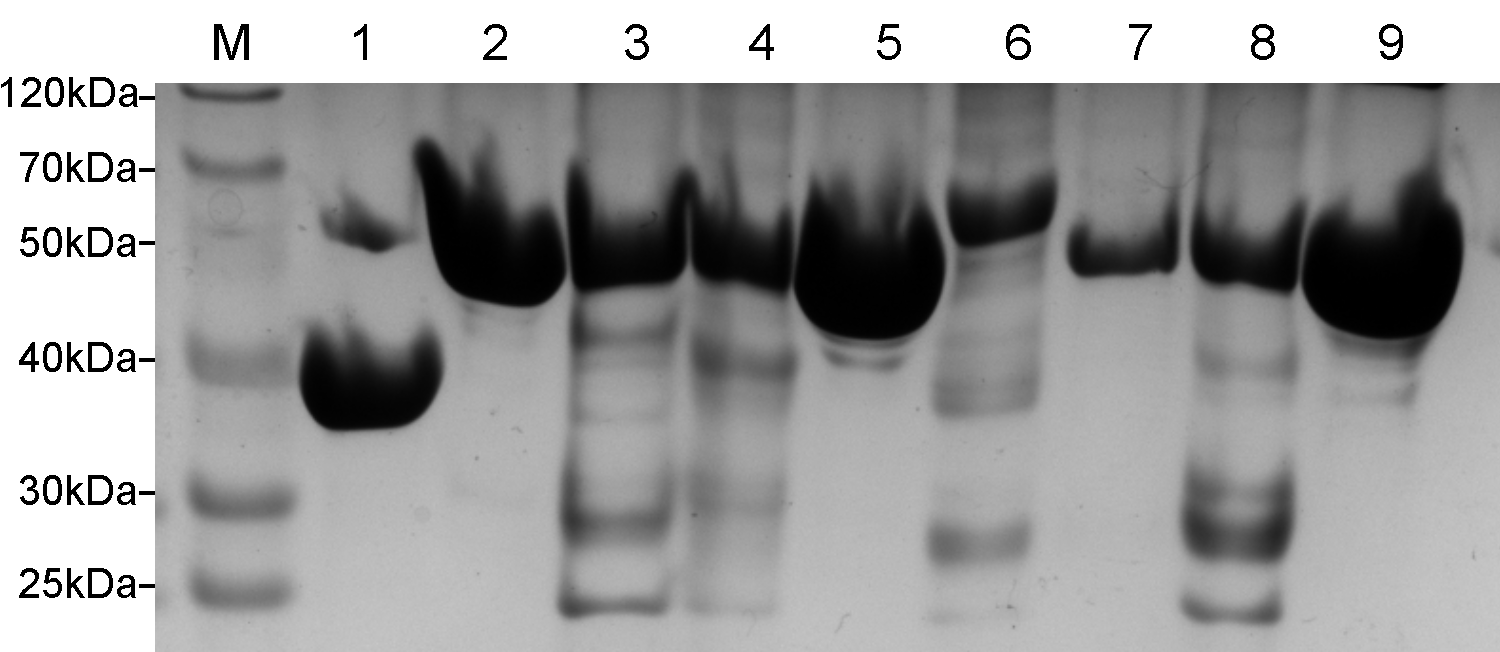

Supplement: Supplementary file 1 [file ijms-24-03421-s001.zip › Figure S2.tif]

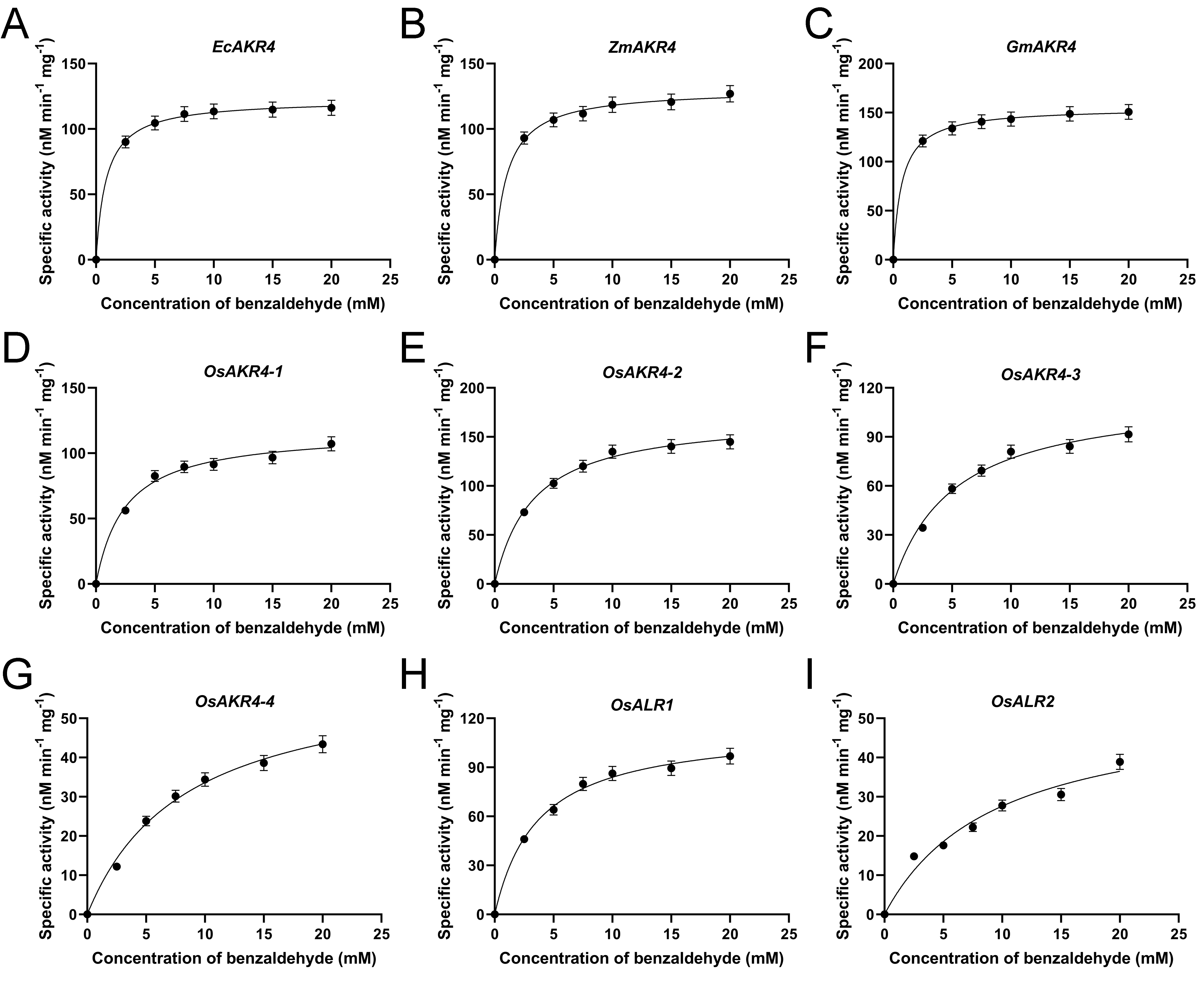

Supplement: Supplementary file 1 [file ijms-24-03421-s001.zip › Figure S3.tif]

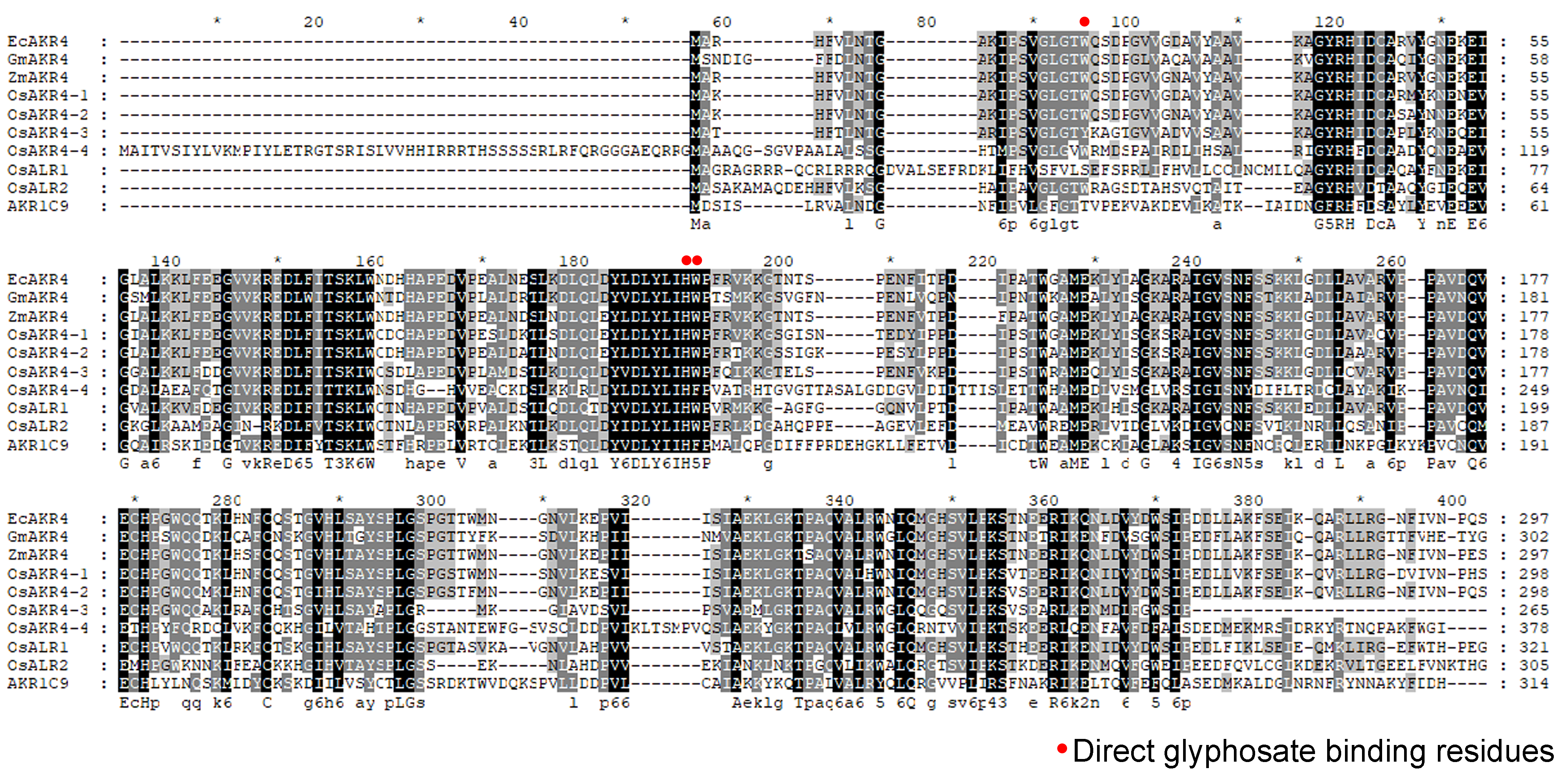

Supplement: Supplementary file 1 [file ijms-24-03421-s001.zip › Figure S4.tif]

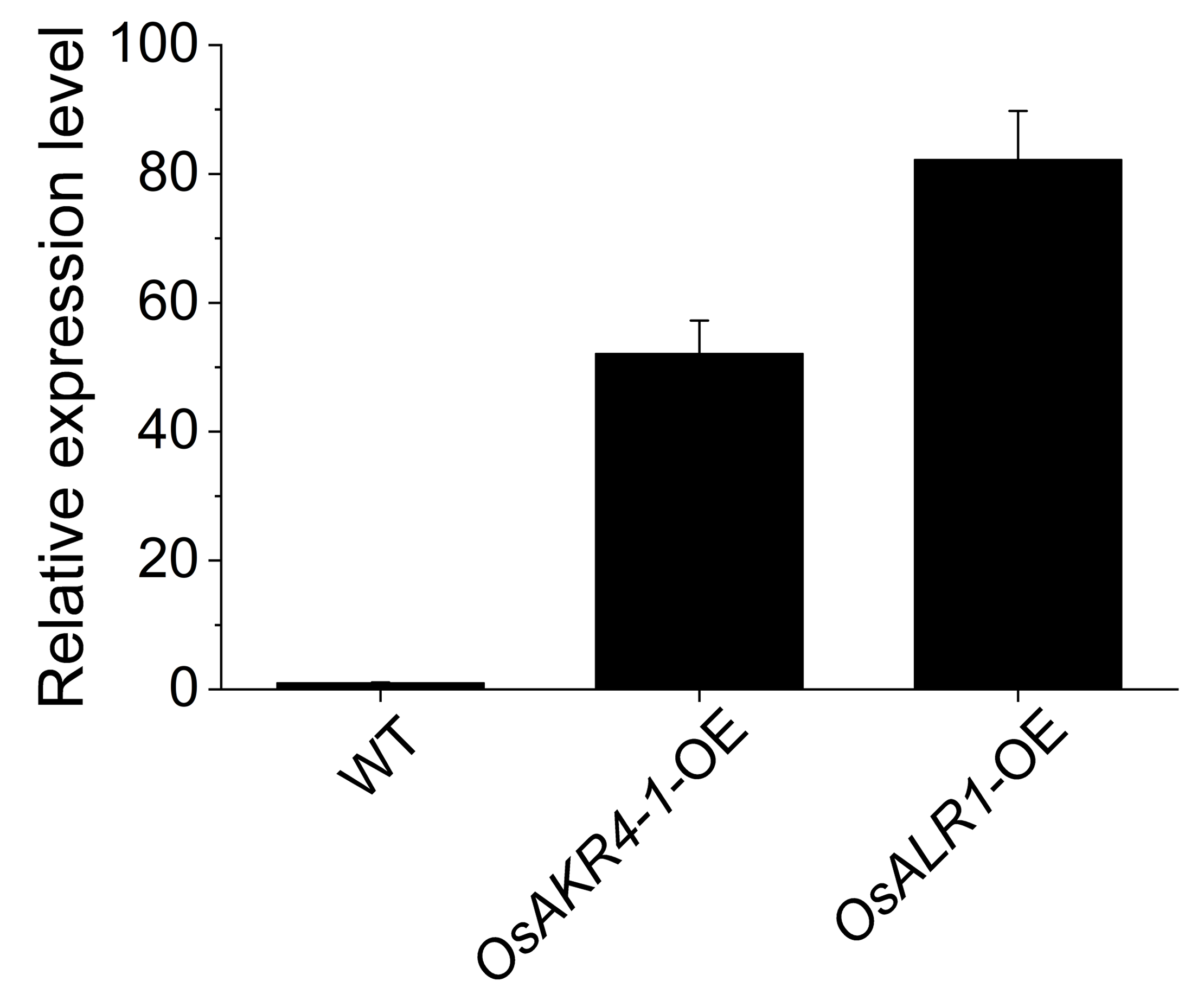

Supplement: Supplementary file 1 [file ijms-24-03421-s001.zip › Figure S5.tif]

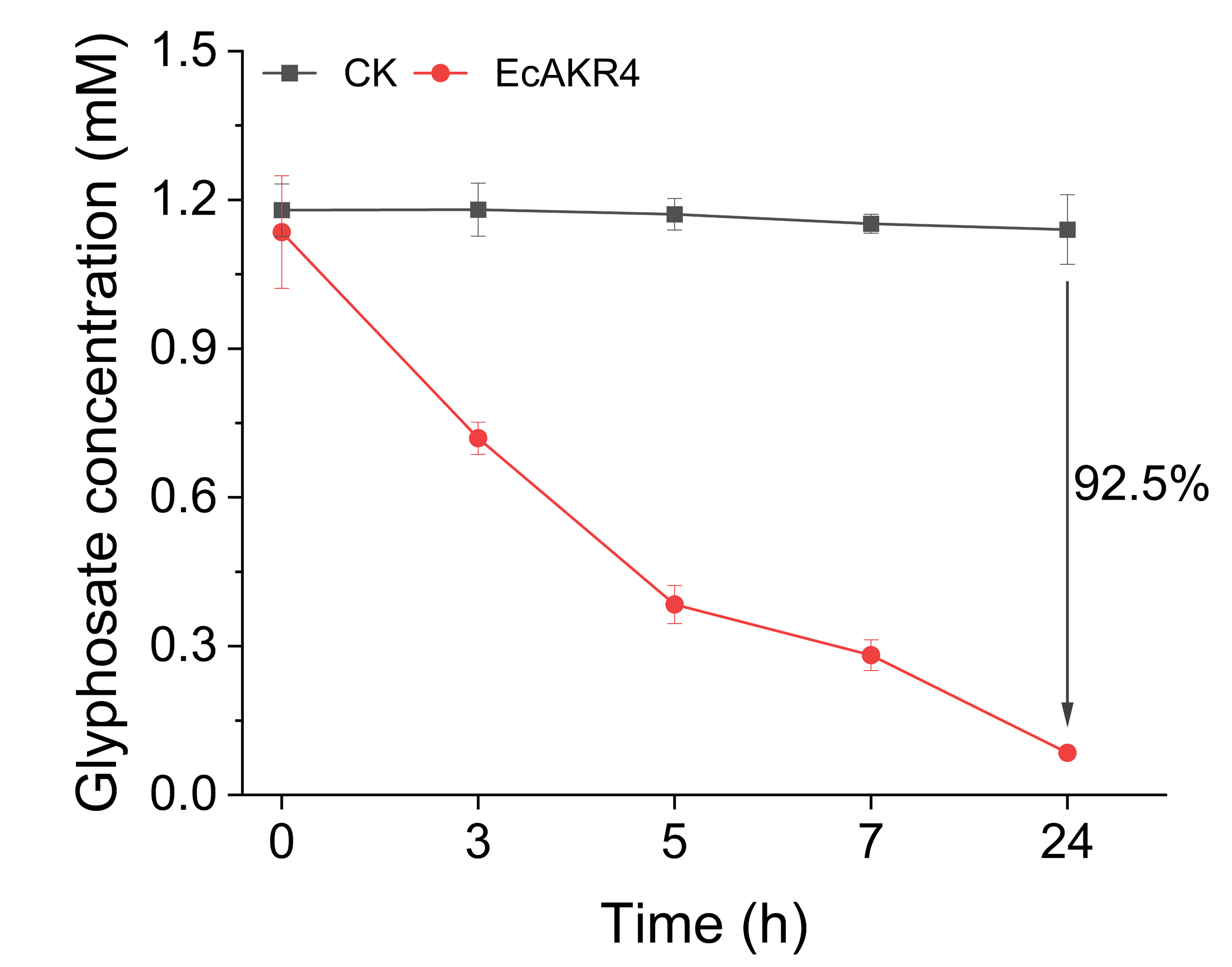

Supplement: Supplementary file 1 [file ijms-24-03421-s001.zip › Figure S6.tif]
